# Airborne lidar-based estimates of tropical forest structure in complex terrain: opportunities and trade-offs for REDD+

**DOI:** 10.1186/s13021-015-0013-x

**Published:** 2015-02-03

**Authors:** Veronika Leitold, Michael Keller, Douglas C Morton, Bruce D Cook, Yosio E Shimabukuro

**Affiliations:** 1 Remote Sensing Division, National Institute for Space Research (INPE), São José dos Campos, SP CEP 12201-970 Brazil; 2 Biospheric Sciences Laboratory, NASA Goddard Space Flight Center, Greenbelt, MD 20771 USA; 3 International Institute of Tropical Forestry, USDA Forest Service, San Juan, 00926 Puerto Rico; 4 EMBRAPA Satellite Monitoring, Campinas, SP CEP 13070-115 Brazil

**Keywords:** Tropical montane forest, Airborne lidar, Digital Terrain Model, Elevation accuracy, Data thinning, Canopy height, Biomass estimation, REDD+

## Abstract

**Background:**

Carbon stocks and fluxes in tropical forests remain large sources of uncertainty in the global carbon budget. Airborne lidar remote sensing is a powerful tool for estimating aboveground biomass, provided that lidar measurements penetrate dense forest vegetation to generate accurate estimates of surface topography and canopy heights. Tropical forest areas with complex topography present a challenge for lidar remote sensing.

**Results:**

We compared digital terrain models (DTM) derived from airborne lidar data from a mountainous region of the Atlantic Forest in Brazil to 35 ground control points measured with survey grade GNSS receivers. The terrain model generated from full-density (~20 returns m^−2^) data was highly accurate (mean signed error of 0.19 ± 0.97 m), while those derived from reduced-density datasets (8 m^−2^, 4 m^−2^, 2 m^−2^ and 1 m^−2^) were increasingly less accurate. Canopy heights calculated from reduced-density lidar data declined as data density decreased due to the inability to accurately model the terrain surface. For lidar return densities below 4 m^−2^, the bias in height estimates translated into errors of 80–125 Mg ha^−1^ in predicted aboveground biomass.

**Conclusions:**

Given the growing emphasis on the use of airborne lidar for forest management, carbon monitoring, and conservation efforts, the results of this study highlight the importance of careful survey planning and consistent sampling for accurate quantification of aboveground biomass stocks and dynamics. Approaches that rely primarily on canopy height to estimate aboveground biomass are sensitive to DTM errors from variability in lidar sampling density.

**Electronic supplementary material:**

The online version of this article (doi:10.1186/s13021-015-0013-x) contains supplementary material, which is available to authorized users.

## Background

Tropical forests are important reservoirs of carbon and biodiversity. Characterizing the spatial distribution of aboveground biomass (AGB) is a prerequisite for understanding carbon cycle dynamics in tropical forests over time. Precise estimates of AGB and changes in carbon stocks from human activities are also required for ongoing climate mitigation efforts to Reduce Emissions from Deforestation and Forest Degradation (REDD+) [1].

Airborne lidar has been successfully used to estimate aboveground biomass in a range of forest ecosystems [2-9]. Typical approaches to predict AGB with lidar data are based on regression models linking lidar metrics to biomass estimates from forest inventory plots. The model is then used to estimate AGB over larger areas. Lidar-derived metrics most frequently used to predict biomass include mean or maximum canopy height [10-13] and vertical canopy profile measures, such as height percentiles and variance of heights [14,15]. Airborne lidar remote sensing supports high-resolution carbon mapping across broad spatial scales and a range of ecosystems [16-18], with great potential to aid carbon monitoring and climate change mitigation efforts (e.g. REDD+).

Estimation of forest canopy height using lidar data depends upon an accurate representation of the ground surface in digital terrain models (DTMs). For forestry studies in particular, lidar is capable of characterizing both terrain and vegetation structure effectively. However, any error in the DTM will propagate to affect the accuracy of the derived vegetation metrics [19] and canopy height models (CHM). Therefore, it is necessary to characterize uncertainties associated with lidar-derived DTMs in order to accurately quantify uncertainties in the overlying vegetation heights.

Ground data are the most common method for estimating the accuracy of lidar-derived elevation estimates. Control points are collected using an independent method with higher accuracy, assuming that the calculated height differences or elevation errors are normally distributed [20]. In this context, and for the purposes of the present study, the quality of the DTM is expressed in terms of vertical accuracy, i.e., how close the lidar-measured terrain elevation is to the *reference value* established from *in-situ* GNSS observations.

The accuracy of lidar-derived DTMs can differ significantly across topographic and land cover gradients. Uncertainty in lidar-derived DTMs encompasses three sources of error: (1) sensor-specific uncertainties associated with the navigation, positioning and lidar systems during data acquisition; (2) geometric uncertainties related to the flight altitude and ranging distance, scan angle, or the local topography; and (3) uncertainties arising during the post-processing steps, such as point classification or surface interpolation [21]. Over open areas with relatively flat terrain, it is common to achieve elevation accuracies below 0.15 m root mean square error (RMSE) [22-24]. In a study evaluating DTM accuracy for six different land-cover types, Hodgson and Bresnahan [25] observed RMSE values ranging from a low of 0.17 to 0.19 m in pavement and low grass classes to a high of 0.26 m in a deciduous forest. In areas covered by dense vegetation, DTM elevation errors tend to increase because less energy reaches the ground, resulting in fewer ground points for DTM surface interpolation [26]. Several studies have assessed lidar-derived DTM accuracy in temperate coniferous, deciduous and mixed forests, reporting RMSE values that range between 0.32 m and 1.22 m [27-29]. However, there have been relatively few studies of elevation accuracy under complex, multilayered tropical rain forest canopies [26] where REDD+ efforts are concentrated.

**Figure 1:**
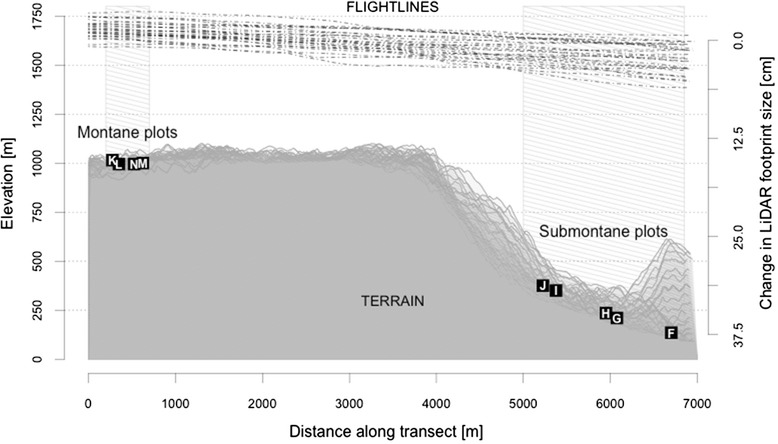
**Graphical overlay of vertical transects along the length of the study area including the 26 individual flightlines and the underlying terrain elevation.** The control points in the montane forest plots (K–N) lie at an average elevation of 1000 m, while those in the submontane plots (F–J) are located between 100–370 m elevation. North is to the left of the figure, and the y-axis on the right-hand side with an inverted scale shows the increase in lidar footprint size with growing distance from the airborne sensor.

In this study, we analyzed 1000 hectares of high-density lidar data collected along a steep elevational gradient (100 m to 1100 m a.s.l.) with coastal Atlantic Forest in Southeast Brazil. Lidar data collection covered nine 1-ha permanent field plots divided between submontane and montane forest areas (Figure 1). We evaluated the accuracy of a DTM derived from the airborne lidar data for the topographically complex study area of the Serra do Mar and assessed the impact of variable survey conditions (i.e. changes in flying height, ranging distance and footprint size) on the characterization of the ground surface. We then assessed how changes in lidar data density influenced DTM accuracy, and examined how DTM uncertainty propagated into lidar-derived canopy height metrics. Our study targeted two main objectives: 1) to provide guidance regarding the minimum lidar point density required to generate DTM accuracies needed for lidar-based studies of forest biomass, and 2) to quantify the impacts of DTM errors on estimates of aboveground biomass. With its complex terrain, steep elevational gradient, and dense multilayered tropical forest canopy, the study site is unlike most of the areas considered in previous lidar forestry studies, but similar to fragments of Brazil’s Atlantic Forest and other tropical forests.

## Results

### Full-density lidar data

Field GNSS elevations and lidar-derived DTM values (1m resolution, full-density data) showed excellent agreement. The error analysis of elevations using all 35 valid control points resulted in a mean signed error of 0.19 ± 0.97 m (μ ± σ), and the calculated RMSE value was 0.97 m. DTM elevations were higher on average than the corresponding GNSS elevations. Considering the uncertainty in calculating the lidar DTM (vertical 1σ = 0.15 m on flat terrain) and error in the GNSS measurements, this 0.19 m elevation difference indicates a very good agreement between field data and the terrain model. Moreover, using only the 30 most accurate control points (σ < 1 m) for comparison, the mean signed error dropped by 63% to 0.07 ± 0.89 m difference of terrain elevations. Based on a one-sided t-test performed with the 30 most accurate control points, the DTM errors were not significantly different from zero (95% confidence level, p-value = 0.662).

DTM accuracy did not differ significantly by forest type or elevation for ground control points collected in submontane and montane forests. Differences between elevation errors associated with submontane and montane areas were evaluated assuming a normal distribution of the errors (Kolmogorov-Smirnov test, p-value = 0.923). Using the 30 most accurate control points for comparison, calculated mean signed errors for submontane vs. montane areas revealed positive differences between DTM and GNSS elevations at lower altitudes (0.23 ± 0.88 m), indicating a slight overestimate from lidar-derived terrain elevations in this area. For montane sites, the difference between DTM and GNSS values was smaller in magnitude and negative (−0.14 ± 0.90 m). However, mean signed errors were not significantly different from each other, based on a two-sided t-test performed with the two sets of errors (95% confidence level, p-value = 0.139). Thus, variability in flying height (footprint size) did not result in a statistically significant difference in DTM accuracy across the study area.

### Reduced-density lidar data

**Figure 2:**
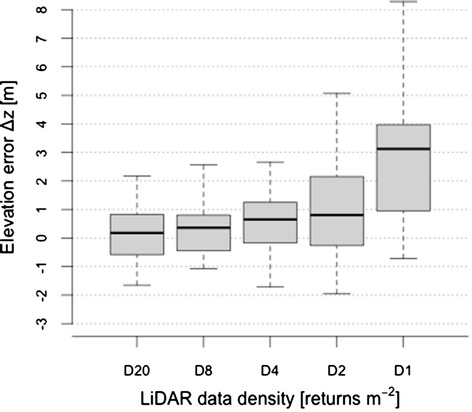
**Distribution of the errors between GNSS and DTM elevations with data density levels of 20, 8, 4, 2 and 1 returns m** ^**−2**^ **(D20, D8, D4, D2 and D1, respectively).**

Lower point densities in the thinned lidar data resulted in less accurate DTMs. Five data density levels were analyzed: the original density of 20 returns m^−2^ (D20) and the thinned return densities of 8, 4, 2 and 1 m^−2^ (denoted D8, D4, D2 and D1, respectively). When compared with GNSS control points, mean signed errors of the thinned DTM elevations increased as data density was reduced from D20 (0.19 ± 0.97 m) to D1 data (3.21 ± 3.12 m) (Figure 2). DTM elevations were higher than the GNSS elevations in all cases, with increasing error magnitudes as data were thinned. Calculated RMSE values showed a similar increasing trend with decreasing data density, ranging from a low of 0.97 m for the D20 DTM to a high of 4.45 m for the D1 data (Table 1).

**Table 1:** **Summary statistics from the DTM error analysis after data thinning**

		**Error statistics (Δz) in meters**				
**Data type**		**Min**	**Max**	**Mean**	**Stdev**	**RMSE**
**D20**	submontane	−1.23	2.18	0.33	0.92	0.95
	montane	−1.65	1.86	0.02	1.02	0.99
	**ALL**	**−1.65**	**2.18**	**0.19**	**0.97**	**0.97**
**D8**	submontane	−2.88	4.51	0.54	1.60	1.65
	montane	−1.07	1.85	0.19	0.90	0.89
	**ALL**	**−2.88**	**4.51**	**0.38**	**1.32**	**1.35**
**D4**	submontane	−1.72	6.98	1.81	2.45	2.99
	montane	−0.94	2.25	0.30	0.97	0.99
	**ALL**	**−1.72**	**6.98**	**1.12**	**2.04**	**2.30**
**D2**	submontane	−1.96	14.62	2.36	3.96	4.52
	montane	−1.39	3.33	0.66	1.32	1.44
	**ALL**	**−1.96**	**14.62**	**1.59**	**3.13**	**3.47**
**D1**	submontane	0.46	14.05	4.42	3.24	5.43
	montane	−0.72	7.49	1.78	2.32	2.87
	**ALL**	**−0.72**	**14.05**	**3.21**	**3.12**	**4.45**

**Figure 3:**
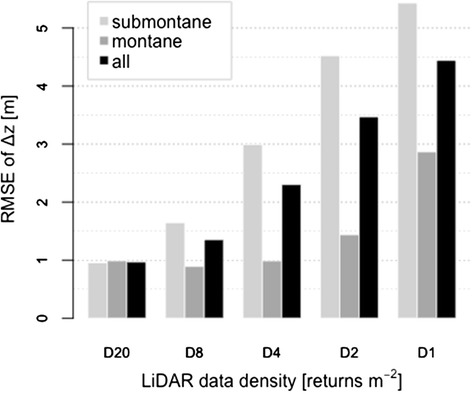
**Comparison of RMSE values in the DTMs based on the five data density levels of 20, 8, 4, 2 and 1 returns m** ^**−2**^ **(D20, D8, D4, D2 and D1, respectively).**

Elevation errors in the thinned DTMs were larger in the submontane region than in the montane area for all the data densities. This observed difference between elevation classes became larger with increased levels of data thinning; the mean signed error difference between submontane and montane areas with 20 returns m^−2^ (0.31 m) increased to 2.64 m when data density dropped to 1 return m^−2^. The trend in RMSE values also followed a similar pattern, with growing differences between submontane and montane DTM accuracy as data were thinned (Figure 3). With the highest data density, submontane and montane RMSE values were nearly identical (<0.1 m difference), while with lower data densities, montane RMSE values remained low while submontane RMSE values increased rapidly (0.76 to 3.08 m difference). The elevation error statistics based on thinned data are summarized in Table 1. These differences likely reflect the combined influence of greater ranging distance and topographic complexity in submontane areas.

**Figure 4:**
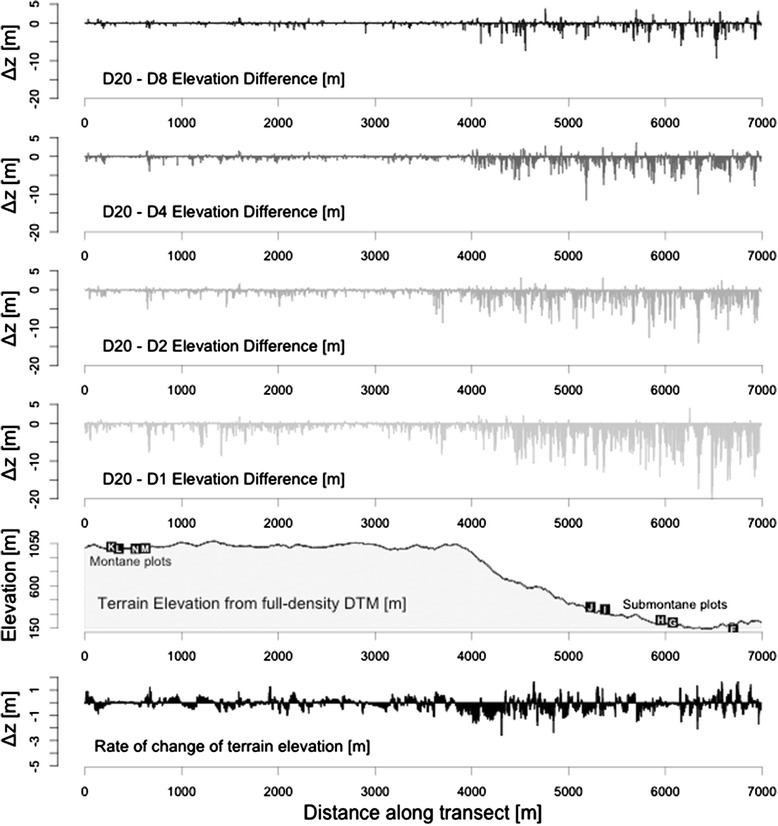
**Elevation differences between the original DTM generated from the full-density data (D20) and the thinned DTMs (D8, D4, D2, D1) extracted from a 1-m grid along the central line of the study area.** A vertical transect of the corresponding terrain elevations extracted from the original DTM along the same central line is shown for reference (note the submontane and montane plot locations), as well as the calculated rate of change of the terrain elevation along the transect.

To illustrate the spatial variability of DTM elevation errors across the landscape, a transect line was drawn along the center of the study area and DTM elevations were sampled from the 1-meter raster grids for all data densities. The difference between the cell values of the full-density DTM extracted along the transect line and the corresponding cell values of each thinned DTM was calculated and the elevation differences plotted (Figure 4). In general, the elevation difference between full-density and thinned DTMs was larger at lower altitudes, along the hillslope and in the valley, and smaller on top of the plateau. The magnitude of the difference increased with increased data thinning throughout the whole area, and the spatial distribution of the errors was associated with the level of complexity of the terrain in all DTMs examined. Where the terrain surface was more accentuated (i.e. greater rate of change of elevation), the corresponding difference in full-density vs. thinned DTM values was also larger, while with a smoother terrain surface, the associated DTM differences were smaller in magnitude.

### Effects of data thinning on estimated canopy height

**Figure 5:**
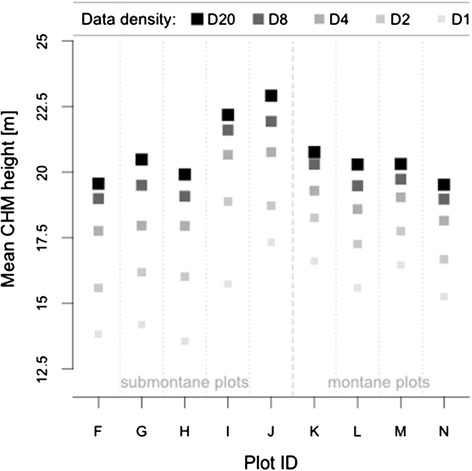
**Mean canopy surface heights associated with the field plot locations (submontane Plots F - J and montane Plots K - L) based on CHMs generated from original and thinned lidar data (D20, D8, D4, D2 and D1 indicate the different data density levels).**

Thinned lidar data consistently underestimated canopy heights in the 1-ha plots (Figure 5). With the full D20 data, mean canopy heights for the nine inventory plots ranged between 19.52 and 22.91 m (Plots F-N). With increasing levels of data thinning, the mean canopy heights decreased on average by 0.70 m (3%), 1.75 m (8%), 3.40 m (16%) and 5.26 m (25%) for return densities of D8, D4, D2 and D1, respectively. The magnitude of canopy height changes was generally larger for the submontane plots (F, G, H, I and J), resulting in mean decreases of 0.79 m, 1.99 m, 3.93 m and 6.08 m with increasing thinning levels. In comparison, the mean canopy height changes in the montane plots (K, L, M and N) was 0.60 m, 1.45 m, 2.73 m and 4.24 m for the return densities of D8, D4, D2 and D1, respectively.

Lidar-derived canopy surfaces (digital surface models, DSMs) at the field plot locations showed little variation with the different levels of data thinning. A visual assessment of the DSM for each plot indicated that the canopy surface became slightly more rugged with increased data thinning, but the overall canopy surface elevation and shape did not change. In comparison, the terrain surface showed larger changes with increased levels of thinning. DTM errors in the thinned datasets resulted from an incorrect classification of vegetation features as ground. The overall effect of thinning was a positive bias in the ground elevation, which translated into lower canopy heights with decreasing data density.

**Figure 6:**
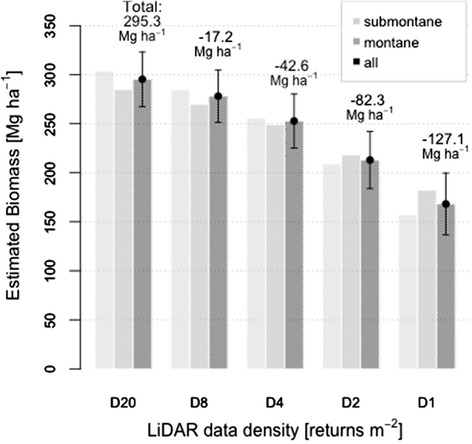
**Aboveground biomass estimates in submontane and montane classes and across all nine permanent plots (mean ± standard deviation) for different data densities predicted with a linear model based on mean canopy surface height.**

Underestimation of mean canopy height (MCH) in the thinned lidar data had a significant impact on modeled aboveground biomass (AGB). We developed a simple regression model for the nine plot locations based on MCH: AGB = 24.13 × MCH - 204.76; r^2^ = 0.43; RMSE = 30.0 Mg ha^−1^. Aboveground biomass predictions (mean ± standard deviation across nine permanent plots) for the different thinning levels ranged from 295.3 (±27.9) Mg ha^−1^ with full-density lidar data to 168.2 (±31.5) Mg ha^−1^ with the lowest data density of 1 return m^−2^ (Figure 6). In this study, a 1–5 m bias in MCH from incorrect ground detection may lead to errors in AGB estimates on the order of 15–125 Mg ha^−1^. For lidar return densities below 4 m^−2^, the bias in height estimates translated into aboveground biomass errors substantially greater than the model error of ~30 Mg ha^−1^. These findings illustrate how approaches that rely on mean canopy height to estimate aboveground biomass are sensitive to DTM errors that arise from variability in lidar sampling density.

## Discussion

### Lidar-derived ground topography

Lidar coverage at the Atlantic Forest study site resulted in a very accurate DTM, despite large elevation differences, steep slopes, and closed canopy tropical forest cover. The ability to generate a highly accurate terrain model in such a challenging environment can be attributed, in part, to the high lidar point density (20 returns m^−2^ on average). Typical lidar data densities used for forest research and management purposes have been within the range of 0.5 - 4 returns m^−2^ [30-32], occasionally reaching a higher value of 10 to 12 returns m^−2^ [33,34]. Our approach to test the impact of data density on DTM accuracy highlights the potential variability in terrain elevations (and therefore canopy characterization) from low-density lidar coverage in regions with complex topography. Thinning of the point cloud below 4 returns m^−2^ led to elevation errors that rendered the resulting DTM inadequate for consistent retrievals of vegetation heights. We therefore recommend a minimum lidar point density of 4 m^−2^ for studies of dense forest vegetation in complex terrain. Dense lidar data coverage is also critical for REDD+ and related applications that require repeat acquisitions to monitor changes in forest structure and aboveground carbon stocks; accurate DTMs are critical for change detection in regions with complex topography.

The results of this study are consistent with previous efforts to validate DTM products from small-footprint lidar systems [27-29], including an exponential increase in errors as data density decreases [35]. Clark and collaborators [26] reported a DTM accuracy of 0.58 m RMSE in open-canopy flat areas of an old-growth Costa Rican rain forest, and overall RMSE of 2.29 m when steep slopes and multilayered dense vegetation areas were also considered. In our study, the lidar-derived DTM consistently overestimated the ground elevation compared to the reference points, likely due to the incorrect classification of vegetation features as ground by the point-filtering algorithm. This overestimation of ground elevation was small in the full density data, but increased with successive thinning of the data.

Importantly, submontane areas consistently showed larger changes in DTM accuracy than montane areas after data thinning – consistent with longer ranging distances, larger lidar footprints, and more complex topography at lower elevations in the study site. Consistent flying altitude for data collection resulted in a change in footprint size as ranging distances increased between montane and submontane areas (Figure 1). Longer ranging distances lower the proportion of pulses that penetrate the forest canopy to generate a return from the ground surface [19,36]. Terrain complexity has been identified as a cause for the variation in DTM accuracy across landscapes [37]. The steeper slopes and more variable topography in the submontane region might be harder to capture by the lidar system than the generally more homogeneous terrain on top of the plateau in the montane forest. Optimization of the flight line configuration at the time of data collection (e.g. constant flying height above ground, even point distribution) could potentially minimize the observed difference between DTM accuracy for areas with different elevations, with important implications for data quality and forest applications.

### Lidar-derived canopy height

The results of this study illustrate how errors in DTM accuracy propagate into estimates of forest canopy structure. Accurate characterization of the ground surface is a prerequisite for lidar vegetation studies because vegetation heights are calculated relative to the associated bare earth surface. Variability in DTM accuracy introduces error in the canopy height calculations, ultimately leading to erroneous estimation of related forest metrics or modeled aboveground biomass. Careful attention to lidar collection and analysis is particularly important in regions with complex topography, given previous issues with large-footprint lidar data in sloped terrain [38] and the relative inaccessibility for field measurements in these sites. Biases that propagate from lidar-derived canopy structure to estimates of aboveground forest biomass on sloped terrain would therefore be less likely to be detected by field validation efforts.

Lidar-based biomass estimates that rely on mean canopy height may be particularly sensitive to height biases from sampling issues that influence the accuracy of the DTM. The consistent overestimation of ground elevation in the analysis of thinned lidar data for Serra do Mar highlights the potential for a directional bias (underestimation of canopy heights) in regions with more sparse lidar sampling. No significant change was observed in the DSM heights with data thinning, suggesting that even with low point density, it is possible to capture the highest points of tree crowns and generate a canopy surface model representative of the true outer vegetation surface. Mountainous areas and other regions with complex topography present unique challenges for uniform lidar sampling. REDD+ efforts at the subnational or national scale will confront these sampling and analytic challenges for forests on steep slopes or other complex terrain. This study provides important guidance on the trade-offs associated with sampling density and biomass estimation over large regions with complex topography.

### Lidar-based biomass estimates

Detailed knowledge of the spatial distribution of aboveground forest biomass is critical to improve estimates of carbon sources and sinks over time. Tropical forest biomass estimates are limited by knowledge of the allometry of tropical trees. The extreme diversity of tree species in tropical forests generally precludes species-specific allometries and instead general relations are applied [39,40]. As in other biomes, quantification of biomass depends on relations between lidar metrics (mainly mean or total canopy height) and estimates of plot biomass from field measurements and allometric equations [41,42]. Lidar-based estimates of forest biomass could greatly improve mapping of aboveground carbon stocks and monitoring carbon emissions over large areas for tropical forests. However, this study suggests caution when applying generalized biomass models based on a single lidar metric (MCH or TCH) across a heterogeneous landscape with both flat and sloped terrain and dense vegetation, like the Serra do Mar, especially at low lidar return densities. Increasing point density mitigates the problem of accurate canopy height (and DTM) generation but increases costs.

Our study points to the need for careful attention to lidar data acquisition parameters to assess aboveground biomass in tropical forests with complex topography. Mascaro and colleagues [43] have called for a global airborne lidar campaign to cover tropical forests. We endorse this proposal but add two important caveats. First, some tropical forest environments will be more costly and complex than others for airborne lidar data acquisition. Additional costs reflect the need to adapt data collection parameters to provide equivalent lidar sampling in domains of simple and complex topography. Wall-to-wall mapping with consistent data collection in montane environments drastically reduces the efficiency of the *fly high and fast* strategy advocated by Mascaro and colleagues. Second, and perhaps more importantly, the legislation controlling airborne lidar survey varies across tropical nations. Brazil contains the largest area of tropical forests of any nation. However, because Brazil has a highly regulated market for aerial survey, achieving pricing as low as estimated by Mascaro and colleagues would be difficult or impossible at present. Regardless of these difficulties, airborne lidar offers a promising avenue for more detailed characterization of the world’s tropical forests – with unique advantages for assessing the spatial and structural complexity of tropical forests in addition to benchmarking forest carbon stocks.

## Conclusions

We found that small-footprint lidar data can be used to characterize the sub-canopy terrain elevation with high vertical accuracy (<1 m) in the topographically complex Serra do Mar region. The accuracy of the lidar-derived ground elevations was more strongly influenced by sampling point density than either the ranging distance or complexity of the terrain features. From the perspective of forest carbon monitoring and REDD+, return densities above 4 m^−2^ are recommended for generating forest structure data for biomass estimation. In addition, we recommend a constant flying height above ground (i.e. equal lidar footprint size), and careful flight planning to generate uniform data density throughout the lidar coverage. A consistent sampling frame is prerequisite for improved lidar-based estimates of aboveground biomass and consistent long-term monitoring under REDD+ and related activities. For dense tropical forests on steep terrain, variability in sampling density and footprint characteristics can introduce large biases in lidar-based estimates of aboveground biomass (up to 80–125 Mg ha^−1^ error in estimated biomass vs. 30 Mg ha^−1^ model error in our case), based on the underestimation of canopy height in areas with low sampling density.

## Methods

### Study area

The study area is located within the São Paulo State Park of Serra do Mar (PESM) (23°34′S and 45°02′W; 23°17′S and 45°11′W) in Southeast Brazil. It is characterized by complex terrain along an altitudinal gradient (0–1200 m a.s.l.) and is covered by the dense vegetation of the Atlantic Forest. The humid tropical forest in this area is subdivided into vegetation types by altitude – lowland, submontane and montane forests – from sea level up to 1200 m elevation [44]. Terrain slope at the study site is steepest at intermediate elevations in the submontane forest areas (200–900 m a.s.l; ~37° average slope), which account for approximately 37% of the study area. The remaining 63% of the study area consists of the relatively flat lowland forests (4.9%) just above sea level (~21° mean slope) and the montane forest region (58.1%) on flatter sites atop the plateau (900–1100 m a.s.l; ~24° mean slope). Our study included nine permanent forest inventory plots that were established along an altitudinal transect in the PESM [45,46]. One plot is located in the lowland forest at an elevation of 100 m (Plot F), four plots in the submontane forest between 180–370 m (Plots G, H, I and J), and four plots in the montane forest at about 1000 m a.s.l. (Plots K, L, M and N). The permanent plots each have a projected area of 1 ha.

### Lidar dataset

Lidar data were collected by the GEOID Ltda. (Belo Horizonte, MG) in April 2012 as part of the Sustainable Landscapes Brazil joint project of the Brazilian Corporation of Agricultural Research (EMBRAPA) and the United States Forest Service (USFS). The study area was overflown with an Optech ALTM 3100 laser scanner instrument at an average flying altitude of 1600 m a.s.l., covering a rectangular strip of the surface (about 1.5 km × 7 km) with a total area of approximately 1000 ha (Table 2). Average pulse density was 12 m^−2^, resulting in an average return density of 20 m^−2^. Aircraft position information for individual flight lines was used to characterize changes in footprint characteristics across the study site. The original lidar data and associated metadata are freely available on the Sustainable Landscapes Brazil Project’s website: http://mapas.cnpm.embrapa.br/paisagenssustentaveis/.

**Table 2:** **Laser system parameters**

**Parameter**	**Specification**
Positioning system	POS AV™ 510 (OEM) - GNSS/L-Band receiver
Horizontal accuracy	≤50 cm (1:1000 scale; PEC “A”); 1σ
Vertical accuracy	≤15 cm; 1σ
System frequency (PRF)	50 kHz
Scan frequency	25 Hz
Scan angle (FOV)	≤20°
Data recording	first/last mode (up to 2 returns per pulse)
Average flight altitude	1600 m a.s.l.
Beam divergence	0.25 mrad (1/e)
Overlap between flight lines	30%

### Lidar processing

Flight line calibration to adjust variables such as heading, roll, pitch and height was performed by the data provider, and the lidar point cloud was processed using the methodology developed by the G-LiHT research group at NASA Goddard Space Flight Center [47]. Height filtering was carried out using a progressive morphological (PM) filter to select ground points from the data set – a critical step for DTM generation from lidar data [37]. The PM filter is used to identify objects in grayscale images based on spatial structure, and works with dilation and erosion in combination with opening and closing operators to separate ground points from non-ground ones [48]. Point classification was followed by Delaunay triangulation to create a triangular irregular network (TIN) of the filtered ground returns, and the TIN was used to interpolate the ground elevations onto a 1-meter raster grid, thus obtaining the DTM [47].

### Lidar thinning

The original lidar point cloud consisted of multiple return data. Data were thinned from the original point density (~20 m^−2^) to four predefined return densities (8, 4, 2 and 1 m^−2^). Thinning was done randomly at 10 × 10 m resolution to achieve the desired point densities. The resulting datasets simulate lower-density lidar coverage. Random thinning reduced the density of returns classified as ground from the full density dataset (D20, 0.289 m^−2^) to 0.113, 0.058, 0.033, and 0.023 for D8, D4, D2, and D1, respectively. Reclassification of ground and canopy returns in the thinned datasets resulted in a larger fraction of points being classified as ground returns after thinning. On average, montane plots had a higher ground point density than submontane plots, but this difference became less apparent with increased levels of thinning.

Full and reduced-density datasets were processed to generate three different data products representing the terrain surface, the canopy heights above ground, and the outer surface of the forest vegetation: Digital Terrain Model (DTM), Canopy Height Model (CHM) and Digital Surface Model (DSM) raster layers at 1-meter resolution. DTM raster grids were created using the G-LiHT methodology, described above. CHM products were also generated using the G-LiHT algorithm by selecting the highest lidar return in every 1-meter grid cell, building a TIN based on these points, and interpolating the canopy heights on a 1-meter raster grid [47]. The DSMs of the outer canopy were produced from only the first-return points in the lidar point cloud using the BCAL LIDAR Tools open-source software package [49].

### Ground data acquisition

Ground survey data collected in June 2013 within the study area were treated as a reference dataset for lidar DTM validation. A total of 36 points were measured under closed forest canopy in the hilly terrain along the altitudinal transect, marking the corner points of the nine permanent forest inventory plots located within the lidar coverage. We used two Topcon HiPer (L1/L2) GNSS receivers, one used as a rover and a second as a base for subsequent differential corrections. These receivers are survey-grade dual-frequency units capable of receiving both NAVSTAR and GLONASS signals. Raw data at the unknown points were collected for 20–35 minutes on average and up to 60 minutes when reception was poor. Base measurements were made at a survey marker (INCRA “ABE M0693”) located at the Santa Virginia station in the PESM, in an open area less than 10 km of the forest plots. Post-processing of the GNSS data was performed to produce the estimated position of the unknown points. Out of the 36 control points, 35 were measured with success, and 30 points had sub-meter accuracy (σ < 1m) in all three coordinates x, y, z (UTM easting, northing and elevation). The remaining 5 points were less accurate (σ < 2.2 m). The GNSS system parameters and measurement conditions during the survey are summarized in Table 3.

**Table 3:** **GNSS system parameters, survey conditions and control points**

**Parameter**	**Specification**
GNSS system	Topcon HiPer L1/L2 receiver
Horizontal accuracy	3 mm + 0.5 PPM
Vertical accuracy	5 mm + 0.5 PPM
System frequency	20 Hz
Linear units	meters
Angular units	degrees
Datum	WGS84
Projection	UTM Zone 23 South
Geoid	MAPGEO 2010
Base Reference Point	INCRA “ABE M0693”
Number of points measured with success	35 (out of 36 total)
Points with σ < 1 m (x,y,z)	30
Points with 1 ≤ σ < 2.2 m (x/y/z)	5
Accuracy (RMSE) Easting	0.006 - 2.130 m; mean = 0.473 m
Accuracy (RMSE) Northing	0.006 - 1.876 m; mean = 0.225 m
Accuracy (RMSE) Elevation	0.019 - 2.195 m; mean = 0.469 m

### Statistical analysis of the datasets

We compared GNSS and lidar DTM elevations for ground reference locations using mean signed error, absolute error, and root mean square error (RMSE) [21]. Mean signed error can be useful to identify the tendency for under- or over-estimation of elevations (i.e. bias), while RMSE represents the overall mean elevation accuracy of a DTM. We note that RMSE has been criticized as a metric for evaluation of DTMs and other map position data [50-52]. However, the criticisms relate to data distributions that deviate strongly from normality. Inspection of Q-Q plots showed no outliers and no obvious deviation from normality, therefore we had no reason to employ alternative metrics. To determine if the difference between the two sets of height points (DTM vs. GNSS elevations) is statistically significant, a two-sided t-test was performed with a confidence level of 95% and assuming a normal distribution of the errors (Kolmogorov-Smirnov test for normality, p-value = 0.923).

Given the significant variation in terrain elevation across the study area (from about 100 m a.s.l. up to 1100 m a.s.l.) and the relatively constant flying altitude during the lidar survey (~1600 m a.s.l.), the sensor height above the ground varied substantially across the 1000 ha lidar coverage (Figure 1). The mean ranging distance between the sensor and the ground surface was ~660 m for the montane region on top of the plateau, while it was about twice as large (~1320 m) for the submontane region. Because of beam divergence, increasing lidar ranging distance results in a larger footprint on the ground. Variation of sensor height above the ground can influence the measurement results, such as laser point density, penetration, ground detection, and calculated metrics [53]. In this study, the lidar footprint diameter doubled between the montane and submontane regions, from ~0.16 m to ~0.33 m, based on the 0.25 mrad beam divergence. To assess the effect of different ranging distances (i.e. variable footprint size) on DTM error across the study area, the control points were grouped into submontane and montane elevational classes, and the error distributions between the groups were compared. To test if the means of the errors associated with the specified elevation classes are statistically different, a two-sided t-test was performed with a confidence level of 95%.

The accuracy of the DTMs generated after data thinning was evaluated using the same approach as with the full-density DTM. Additionally, we assessed the total number of lidar returns and the number of ground returns in the reduced-density point clouds for each permanent field plot location. The ground point density (points m^−2^) and the fraction of ground returns out of all returns (%) was calculated for each thinning level to quantify the change in commission errors resulting from the ground classification algorithm.

Plot-scale lidar metrics and forest inventory data were used to establish lidar-biomass relationships following standard methods. The goal of this effort was to assess the impact of DTM errors from variability in sampling density on predicted aboveground biomass. We used forest inventory data from the Serra do Mar permanent plot network (Biota Project, see [46]) to calculate field-based AGB estimates in the nine plots following the methodology applied by Alves and colleagues [45]. A linear model was developed to predict AGB based on plot-level mean canopy surface heights derived from the full-density lidar data. We used this regression equation to generate biomass estimates based on the thinned lidar datasets with mean canopy surface height as the predictor, and compared the resulting values across the different data densities.

## Abbreviations

AGB: Aboveground biomass
CHM: Canopy height model
DSM: Digital surface model
DTM: Digital terrain model
LiDAR: Light detection and ranging
MCH: Mean canopy height
TCH: Top-of-canopy height
TIN: Triangular irregular network
REDD: Reduced emissions from deforestation and forest degradation
RMSE: Root mean square error
